# Asymptomatic SARS Coronavirus Infection among Healthcare Workers, Singapore

**DOI:** 10.3201/eid1107.041165

**Published:** 2005-07

**Authors:** Annelies Wilder-Smith, Monica D. Teleman, Bee H. Heng, Arul Earnest, Ai E. Ling, Yee S. Leo

**Affiliations:** * Tan Tock Seng Hospital, Singapore;; † National Healthcare Group, Singapore;; ‡ Singapore General Hospital, Singapore

**Keywords:** asymptomatic SARS Coronavirus infection, SARS seroconversion, SARS epidemiology, Singapore

## Abstract

We conducted a study among healthcare workers (HCWs) exposed to patients with severe acute respiratory syndrome (SARS) before infection control measures were instituted. Of all exposed HCWs, 7.5% had asymptomatic SARS-positive cases. Asymptomatic SARS was associated with lower SARS antibody titers and higher use of masks when compared to pneumonic SARS.

The patterns of spread of severe acute respiratory syndrome (SARS) suggest droplet and contact transmission (1,2). Close proximity of persons and handling of human secretions (respiratory secretions, feces, and the like) enhance the risk for transmission. These facts, together with the fact that transmission is more likely in more severely ill people (who end up in hospitals), have made the hospital setting particularly vulnerable to the rapid amplification of SARS (1–4).

Singapore was one of the countries most affected in the worldwide outbreak of SARS, with a total of 238 cases (available from http://www.who.int/csr/sars/country/table2003_09_23/en/); 76% of infections were acquired in a healthcare facility. The clinical spectrum of SARS has a strong predominance towards more severe disease associated with pneumonia (5); reports on the incidence of asymptomatic or mild infections attributable to SARS-associated coronavirus (CoV) were conflicting (5–8).

We conducted a seroepidemiologic cohort study among healthcare workers (HCWs) exposed to SARS patients in the first month of the nosocomial SARS outbreak at Tan Tock Seng Hospital in Singapore. Our study goal was to investigate the incidence of and factors associated with asymptomatic SARS-CoV infection.

## The Study

Three patients with SARS were admitted to 3 wards of the hospital in early March 2003, at a time when SARS was not recognized and no infection control measures were in place. Patient 1, who had imported SARS from Hong Kong, was admitted on March 1 and isolated after 5 days. Patient 2, a nurse who had looked after patient 1, was initially misdiagnosed as having dengue and was isolated 3 days after her admission when SARS was suspected. Patient 3 was admitted for other reasons (septicemia, ischemic heart disease, diabetes) but shared a cubicle with patient 2, became infected, and was not isolated until 8 days later, since initially the diagnosis of SARS was not considered (3). From March 6 onwards, HCWs were using N95 masks, gowns, and gloves for personal protection when nursing patient 1 and any persons suspected of having SARS. This meant that when providing nursing care for patients 2 and 3, HCWs did not use personal protective measures until SARS was suspected and the suspected patients were isolated. By March 22, N95 masks, gowns, and gloves were mandatory for all HCWs for any patient contact in the hospital.

Information on staff working on these 3 wards during March 1–22 was retrieved from the outbreak investigation team at the hospital and Human Resources. Only HCWs with exposure to any of these 3 patients were included. Exposure was defined as contact with any of these 3 patients in the same room or cubicle. Telephone interviews were conducted in April 2003, using a closed questionnaire by staff experienced in epidemiologic investigations from the hospital's Department of Clinical Epidemiology. Information collected included demographic data (age, sex, and ethnic group), occupation, history of medical conditions, and history of performing procedures with transmission risk (date, place, type, duration, and frequency). Contact time was defined as the total time in the same room with l of the 3 patients. Study participants were surveyed on their use of personal protection, i.e., wearing of N95 masks, gloves, and gown, and consistent handwashing. To verify exposure, names of source patients were included in the questionnaire, and respondents were asked if they had cared for these patients or been close to them (within the same room). Those without direct exposure were excluded from the study. Venous serum samples were taken in May and June 2003, 8–10 weeks after exposure, after informed written consent was given. Serum samples were tested serologically for SARS-CoV total antibodies by enzyme-linked immunosorbent assay (ELISA) using SARS-CoV–infected Vero E6 cell lysate and uninfected Vero E6 cell lysate supplied by the Centers for Disease Control and Prevention (9). The conjugate used was goat antihuman immunoglobulin (Ig)A, IgG, and IgM conjugated to horseradish peroxidase (Kirkegaard & Perry Laboratories, Inc., Gaithersburg, MD, USA). Samples positive for SARS were repeated again and then confirmed by use of an indirect immunofluorescence assay. The specificity of our ELISA was 100%, as tested in 50 serum samples from patients admitted to a non-SARS hospital for illnesses other than respiratory problems: we tested both IgG and IgG; all were negative. Samples from all initially positive patients during the SARS outbreak were sent to the National Environment Agency, Singapore, for confirmation with a neutralization test, and we found a good correlation (data not shown). Laboratory personnel were blinded to the clinical data.

Patients with a positive SARS serologic result, fever, respiratory symptoms, and radiologic changes consistent with pneumonia were defined as having pneumonic SARS. SARS-CoV–positive patients with fever and respiratory symptoms without radiologic changes were defined as having subclinical (nonpneumonic) SARS. SARS-CoV–positive patients without fever or respiratory symptoms were defined as having asymptomatic SARS-CoV infection. The study was approved by the Ethics Committee of Tan Tock Seng Hospital.

A total of 105 HCWs were identified by the outbreak team; 98 (93%) consented to answer the questionnaires, and 80 of these 98 (82%) also consented to have SARS serologic tests performed. Those who had SARS serologic tests did not differ from those who did not have these tests in terms of age, sex, job, or contact time.

The median age of the 80 study participants was 28 years (range 19–64), and 73 (91%) were female. Eight were doctors, 62 were nursing staff (staff nurses, assistant nurses, and healthcare assistants), and 10 had other occupations (cleaners, radiology technicians, physiotherapists). All reported to have had contact with 1 of the 3 index SARS patients. Distance to the source patient was <1 m in 73 cases (91%) and >1 m in 7 cases (9%). All 3 index cases resulted in a similar number of secondary cases (range 10–18 secondary cases).

Of these 80 hospital staff, 45 (56%) were positive by SARS serology. Of the 45 SARS-CoV–positive study participants, 37 (82%) were classified as having pneumonic SARS, 2 (4%) as having subclinical SARS, and 6 (13%) as having asymptomatic SARS-CoV infection (Table 1). Four staff members had fever and cough but negative SARS serologic test results; none of them was diagnosed as having suspected SARS by the hospital's SARS outbreak team. The overall incidence of asymptomatic SARS-CoV infection was 6 (7.5%) of 80. The incidence of SARS-CoV–positive cases among all asymptomatic HCWs was 6 (16%) of 37. The median titer of SARS antibodies was 1:6,400 (range 1:1,600–1:6,400) for pneumonic SARS, 1:4,000 (range 1:1,600–1:6,400) for subclinical SARS cases, and 1:4,000 (range 1:400–1:6,400) for asymptomatic cases (Table 1). The antibody titer for the asymptomatic cases was significantly lower than that for the pneumonic SARS cases (Mann-Whitney test; p = 0.0128). On univariate analysis, sex, age, use of gloves, handwashing, contact with l of the initial 3 patients, distance to the patient, and contact time were not associated with asymptomatic SARS. However, a higher proportion of those who had asymptomatic SARS (50%) had used masks compared to those in whom pneumonic SARS developed (8%) (p = 0.025) (Table 2).

**Table 1:** Clinical spectrum of SARS-CoV–positive cases and SARS antibody titers*

Classification	No. (%)	Median titer (range)
Pneumonic SARS	37 (82.2)	1:6,400 (1:1,600–1:6,400)
Subclinical (nonpneumonic) SARS	2 (4.4)	1:4,000 (1:1,600–1:6,400)
Asymptomatic SARS	6 (13.3)	1:4,000 (1:400–1:6,400)

*SARS, severe acute respiratory syndrome. CoV, coronavirus.

**Table 2:** Univariate analysis of risk factors associated with asymptomatic versus pneumonic SARS*†

Variable	Asymptomatic SARS	Pneumonic SARS	p value‡	Controls	p value§
Median antibody titer (range)	1: 4,000 (1:400–1:6,400)	1:6,400 (1:1,600–1:6,400)	0.013¶	NA	NA
Mean age (SD)	26.5 (4.3)	29.6 (9.2)	0.706¶	33.7 (11.5)	0.098#
Females (%)	6 (100)	32 (86)	>0.999	49 (94)	0.321
No. who used masks (%)	3 (50)	3 (8)	0.025	21 (40)	0.002
No. who used gloves (%)	1 (17)	10 (26)	>0.999	24 (46)	0.090
No. who washed hands (%)	4 (67)	29 (76)	0.63	47 (90)	0.110
No. who were close to a SARS patient (≤3 ft), %	5 (83)	35 (92)	0.456	48 (92)	0.747
Median contact time in minutes (range)	67.5 (10–360)	60 (10–480)	0.863¶	30 (10–960)	0.879#

*SARS, severe acute respiratory syndrome; NA, not available. †SARS serology–negative asymptomatic controls added for comparison. All p values from Fisher exact test or chi-square test, unless otherwise stated. ‡p value for comparing asymptomatic versus pneumonic SARS. §p value for comparing any 2 pairs in the 3 groups. For multiple comparisons, level of significance was set at 0.017 using the Bonferroni method. ¶p values from Mann-Whitney test. #p values from Kruskal-Wallis test.

## Conclusions

We found a substantial number of cases with asymptomatic SARS-CoV infection and subclinical (nonpneumonic) SARS during the initial outbreak of SARS at Tan Tock Seng Hospital in Singapore: the incidence of asymptomatic cases among all exposed HCWs was 7.5%, and the proportion of asymptomatic cases out of all SARS-CoV–positive cases was 13%. Our findings regarding asymptomatic or subclinical SARS-CoV–positive HCWs contradict results from some previous studies, which reported an absence of asymptomatic SARS cases (5–7), but agree with results from other studies (8,9). Our incidence rate of 7.5% was higher (although not significantly) than that of 3% and 2.3% reported in asymptomatic HCWs who cared for SARS patients in Hong Kong (8,10). This difference is most likely due to the greater extent of exposure: a large proportion of our cohort was in close, unprotected contact to SARS patients before infection control measures were in place. However, direct comparison is not possible as the exposure is not described in the Hong Kong cohort. The extent of exposure in our cohort also contributed to the high attack rate that we observed (57%). False positivity may have also played a role but is unlikely or minimal given the high specificity of our essay and reports of high specificity from other centers (5,8,11). Overall, a rate of 13% for asymptomatic SARS cases among all SARS-positive cases is lower than the rate of asymptomatic cases of many other viral respiratory diseases. This difference may be explained by the novelty of this emerging pathogen. Because of its minimal genetic relatedness to other coronaviruses of humans and animals, lack of cross-protective immunity may be associated with development of overt disease (5). However, 1 study reports that subclinical SARS-CoV infections may be more common than SARS-CoV pneumonia, when a sensitive ELISA for SARS-CoV is used (8).

We investigated differences between asymptomatic SARS-CoV infection and pneumonic SARS. We found no difference between pneumonic SARS patients and asymptomatic SARS-CoV–positive patients in relation to age, duration and distance of exposure to source patients, handwashing, and use of gloves. These findings indicate that HCWs who are exposed to SARS can be infected with SARS, regardless of the intensity of exposure. However, mask use was significantly more common in asymptomatic SARS-positive versus pneumonic SARS-positive patients. Antibody titers against SARS-CoV were significantly lower in those who remained asymptomatic, consistent with reports from Hong Kong (12). The person with the lowest SARS antibody titer in our cohort was the only one who had only indirect contact with 1 of the 3 initial patients, and she has remained asymptomatic. These observations suggest that the extent of exposure to SARS in persons who remained asymptomatic may have been lower, possibly resulting in a lower viral load of SARS-CoV, associated with less severe symptoms. A correlation with viral load and disease severity has been suggested (13); however, this hypothesis remains controversial as the development of severe respiratory distress is also thought to be due to an overwhelming immunologic response (1,4). Higher viral loads have been associated with increased severity in some but not all viral diseases. Any association between the infecting dose of SARS-CoV and severity of disease needs to be confirmed with animal studies. The low antibody levels observed in asymptomatic SARS-positive cases could also be because asymptomatic patients do not mount as much of an antibody response. It is also possible that cross-reactive antibodies were measured, although this is unlikely given the high specificity of our assay. The existence of asymptomatic or subclinical cases has public health implications, as they may either serve as a reservoir or as an unknown source of transmission. If asymptomatic persons contribute substantially to transmission but are not readily identified as having SARS, control measures will be hampered since they depend on the ready identification of persons who have been exposed to definite cases (14). Based on our data in Singapore, transmission from asymptomatic patients appears to play no or only a minor role, as all but 1 of the pneumonic cases of SARS had a definitive epidemiologic link to another pneumonic SARS contact. Lack of transmission from asymptomatic patients was also observed in other countries with SARS outbreaks (1; http://www.who.int/csr/sars/en/WHOconsensus.pdf, 2003). As the survival ability of SARS-CoV in human specimens and in environments seems to be relatively strong (15), determining whether asymptomatic patients excrete SARS-CoV is important. We were unable to determine this in our cohort, since these cases all occurred at a time when even the causal agent of SARS was not yet known and no diagnostic tests were available.

We documented a substantial incidence of asymptomatic SARS-CoV infection in exposed healthcare workers before full infection control was in place. Asymptomatic SARS-CoV infection was associated with lower SARS antibody titers and better protective measures (masks) compared to pneumonic SARS.
